# Low sidelobe silicon optical phased array with Chebyshev amplitude distribution

**DOI:** 10.1515/nanoph-2023-0507

**Published:** 2024-01-22

**Authors:** Shi Zhao, Daixin Lian, Wenlei Li, Jingye Chen, Daoxin Dai, Yaocheng Shi

**Affiliations:** State Key Laboratory for Modern Optical Instrumentation, Center for Optical & Electromagnetic Research, International Research Center for Advanced Photonics, College of Optical Science and Engineering, Zhejiang University, Zijingang Campus, Hangzhou 310058, China; State Key Laboratory for Modern Optical Instrumentation, Center for Optical and Electromagnetic Research, International Research Center for Advanced Photonics, Ningbo Innovation Center, College of Optical Science and Engineering, Zhejiang University, Hangzhou, China; Jiaxing Key Laboratory of Photonic Sensing & Intelligent Imaging, Jiaxing 314000, China; Intelligent Optics & Photonics Research Center, Jiaxing Research Institute Zhejiang University, Jiaxing 314000, China; Ningbo Innovation Center, Zhejiang University, Ningbo 315100, China

**Keywords:** silicon photonics, optical phased array, low-sidelobe arrays

## Abstract

We propose and demonstrate a silicon photonic optical phased array (OPA) with ultra-low sidelobe level. The arbitrary ratio power splitters (ARPSs) are introduced to manipulate the amplitude distribution between different channels and suppress the sidelobe level. A 32-channel OPA has been designed and demonstrated with the amplitude distribution determined by preferred Chebyshev method. The experimental results indicate that the sidelobe suppression ratio (SLSR) can be up to 25.3 dB. The measured field of view (FOV) is 84° × 13° with divergence of 2.8° × 1.7°. Furthermore, the frequency-modulated continuous-wave (FMCW) based ranging has been also demonstrated experimentally by utilizing the OPA as the transmitter.

## 1 Introduction

The silicon-on-insulator (SOI) platform is promising for many applications, due to its CMOS compatibility and high integration density, including light detection and ranging (LiDAR). The silicon integrated optical phased array (OPA), an all-solid-state beam steering scheme, has attracted much attention with the advantages of high stability, fast scanning speed, and compact size [1]. Many investigations have been reported on enlarging the field of view (FOV) [2], [3], [4], [5], improving the resolution [6], [7], [8], [9], and reducing the power consumption [10], [11]. Generally, the far-field intensity pattern of the conventional OPA is sinc^2^ function type because of the uniform aperture field amplitude distribution [12]. The sidelobe suppression ratio (SLSR) is then limited to 13 dB theoretically. Recently, researchers begin to focus on achieving higher SLSR [13], [14], [15]. The sparse array is a method to achieve high SLSRs. Sidelobe level of a sparse array can be reduced as the number of elements increases but achieving high SLSR needs significant number of array elements [3], [15], [16]. Manipulating the amplitude distribution of the antenna array is an efficient way to improve the SLSR without requirements for large number of array elements. The star couplers are commonly used for power splitting with Gaussian distribution, and SLSRs of 16 dB [13] and 19 dB [11] have been achieved experimentally. However, the distribution form other than Gaussian is difficult to realize due to the low degree of freedom of star couplers. In Ref. [14], the Gaussian distribution is also achieved by using the cascaded directional couplers (DCs), and the SLSR is measured to be 15.1 dB. However, DCs usually suffer from small bandwidth and low fabrication tolerance. High fabrication precision is required to ensure the precise control of coupling strength and avoid deterioration of the sidelobe suppression.

In this work, we propose an arbitrary amplitude distribution integrated circuit and demonstrate the Dolph-Chebyshev amplitude distribution for the first time. A 32-channel OPA has been designed and fabricated with an antenna pitch of 1.2 μm. Due to the fabrication-friendly arbitrary ratio power splitters (ARPSs), the amplitude distribution among the channels can be well agreed with the simulation. Experimental results indicate that the SLSRs can reach 25.3 dB, and the average SLSR in the FOV is 19.1 dB. The FOV is measured to be 84° × 13° with the divergence of 2.8° × 1.7°. Moreover, we apply the OPA emitter to the frequency-modulated continuous-wave (FMCW) system and demonstrate light ranging experimentally.

## 2 Design and analysis

**Figure 1:**
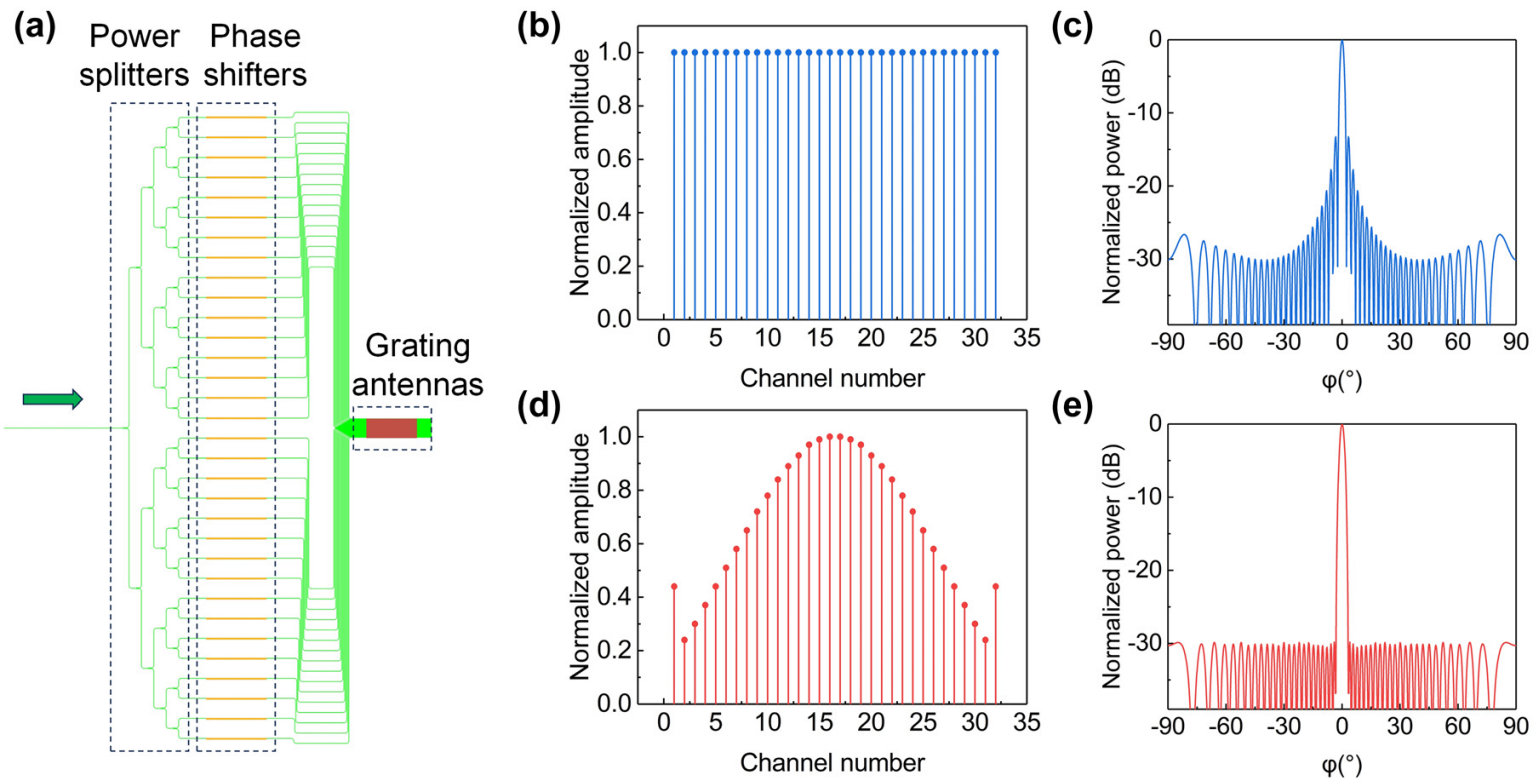
Illustration of  low sidelobe optical phased array. (a) Schematic of the proposed 32-channel OPA with low sidelobe level; (b) uniform amplitude distribution; (c) the far-field pattern of (b); (d) Dolph-Chebyshev amplitude distribution; (e) the far-field pattern of (d).

Figure 1(a) shows the schematic of the proposed 32-channel OPA with low sidelobe level, which consists of the beam splitter tree, the phase shifter array, and the grating antenna array. The light is injected from the input port and divided into each phase shifter element by the beam splitter tree, and then emitted to free space via the grating antenna array. Each channel has an independent controlled phase shifter. The beam steering in the azimuthal (*ϕ*) direction is achieved by controlling the phase front across the grating antenna array. The far-field pattern, described by Fraunhofer diffraction theory, is basically the complex Fourier transform of the aperture field distribution [17]. The conventional OPAs, utilizing uniform 3 dB power splitters (e.g., multimode interferometers, MMIs), have the same amplitude among each channel in the aperture field distribution, which can be regarded as applying a rectangular window, as shown in Figure 1(b). The far-field power distribution calculated using the Fourier transform is the sinc^2^ pattern as shown in Figure 1(c). The maximum sidelobe is the first sidelobe with SLSR of about 13 dB. The SLSRs can be improved by manipulating the amplitude distribution to avoid abrupt truncation of amplitude at the edges. We choose the Dolph-Chebyshev amplitude distribution [18] in this work, which has been widely used in wireless communication [19], [20], [21] and is also named the optimal distribution [22]. Under the given aperture size, it has the following characteristics: (1) same sidelobes level in 180° FOV; (2) achieving the minimum sidelobe level with a given beam width; (3) achieving the narrowest beam width with a given sidelobe level. Hence, the Dolph–Chebyshev amplitude distribution is preferred than Gaussian distribution. The amplitude distribution can be calculated using Chebyshev polynomials when giving the SLSR and channel number (part 1, Supplementary). Here, we set SLSR as 30 dB. The amplitude distribution is shown in Figure 1(d) and the corresponding far-field distribution is shown in Figure 1(e).

**Figure 2:**
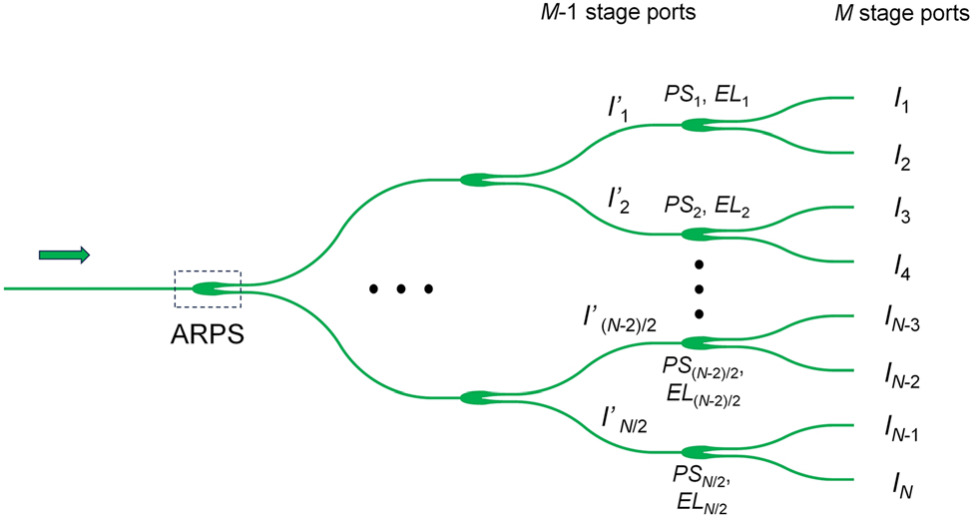
Schematic of the beam splitter tree.

The amplitude distribution depends on the beam splitter tree. We use ARPSs to form the beam splitter tree. The design and tolerance analysis of ARPSs are shown in part 2, Supplementary. An inverse designed method is proposed to calculate the required splitting ratio for the beam splitter tree according to the given amplitude distribution. Figure 2 shows the schematic of the splitter tree with *N* output ports. The intensity distribution at the last stage (*M*) ports is (*I*_1_, *I*_2_, …, *I*_*N*−1_, *I*_*N*_). The power splitting ratios of the connected power splitters can be expressed as:

(1) $$PS_{\text{i / }2}\;=\;\frac{I_{i}}{I_{i-1}+I_{i}}\;\left(i=2,4,\ldots ,N\right)$$

Then, the corresponding device parameters as well as excess loss (*EL*) can be obtained, which has been developed in our previous work [23]. For the *M* − 1 stage ports, the intensity distribution could be calculated using the following equation:

(2) $${I^{\prime }}_{i/2}\;=\;\left(I_{i-1}+I_{i}\right)\cdot 10^{\left(\frac{EL_{i/2}}{10}\right)}\;\left(i=2,4,\ldots ,N\right)$$

Benefiting from the low loss, fabrication-friendly, and easy design characteristics of the ARPSs, the splitter tree is flexible and arbitrary amplitude distribution could be achieved theoretically. The ARPSs parameters of the whole splitter tree can be calculated step by step (Figure S8, Supplementary).

The beam steering in polar (*θ*) direction is achieved by wavelength tuning. The beam steering angle *θ* is determined by the grating equation [1], shown as follows:

(3) $$\theta =sin^{-1}\left(n_{\mathrm{eff}}-\frac{\lambda }{\Lambda }\right)$$

where *n*_*eff*_ is the effective index of the guiding mode, *λ* is the working wavelength and *Λ* is the grating period of antenna. Thus, two-dimensional (2D) beam steering could be obtained by combining phase tuning and wavelength tuning.

## 3 Fabrication and measurement

**Figure 3:**
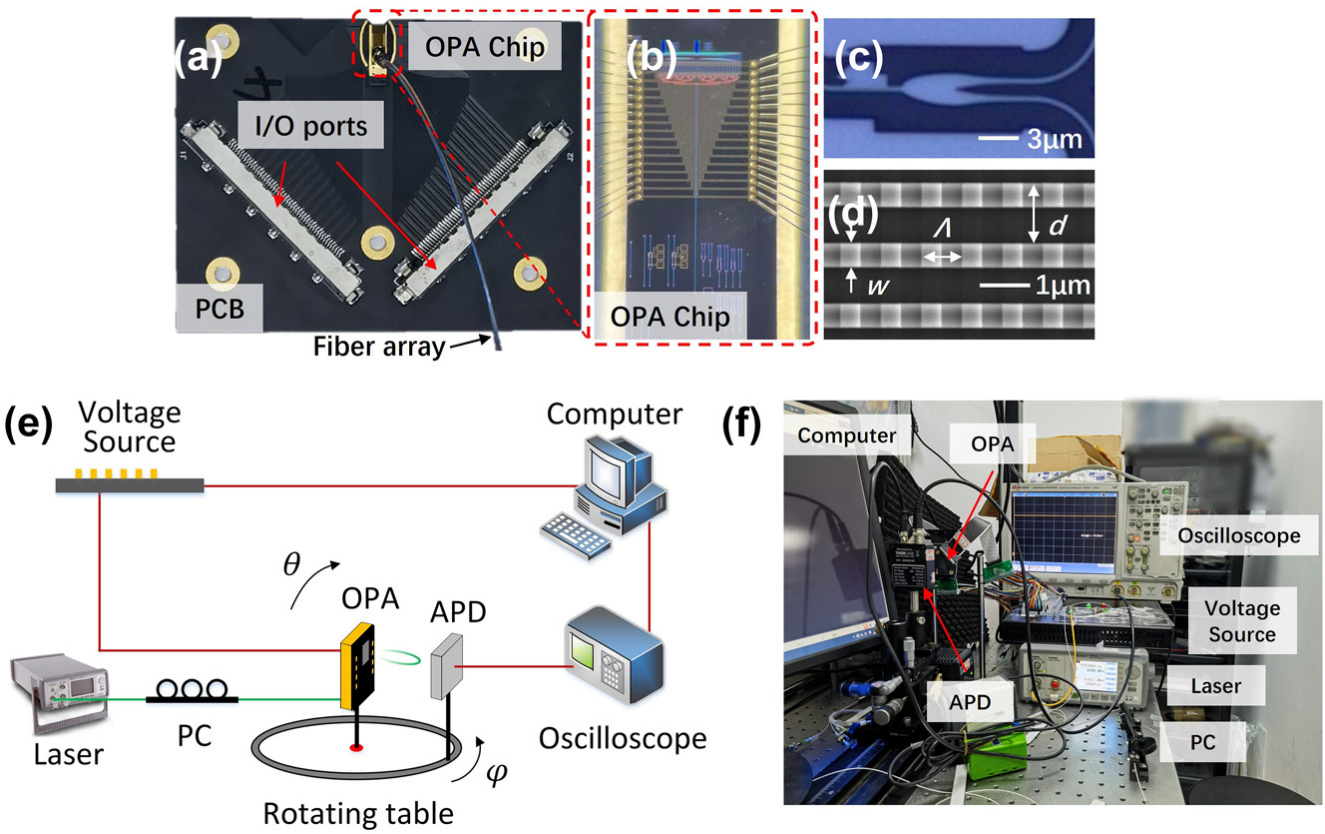
Optical microscopy images of (a) the packaged chip, (b) the fabricated OPA chip, and (c) ARPS; (d) SEM image of grating antennas; (e) schematic and (f) picture of the far-field measurement setup.

The proposed OPA is fabricated on the 220 nm SOI platform with 2 μm buried oxide layer and 1.8 μm cladding oxide layer. The phase shifters are based on thermo-optical effect. The metal heaters, with width of 3 μm and length of 100 μm, consist of 20 nm chrome (Cr) and 200 nm titanium (Ti). The average resistance is measured to be 270 Ω. The distance of adjacent phase shifters is set as 40 μm. The thermal crosstalk is analysed in part 5, Supplementary. The two ends of the heaters are connected to the pads, and then wire-bonded with the printed circuit board (PCB), as shown in Figure 3(a) and (b). The multi-channel voltage source is connected to the PCB through the I/O ports and independently controls the phase shift of each channel. The light couples into the chip as TE_0_ mode by a TE-type grating coupler. The fiber array is packaged on the chip to facilitate measurement. Figure 3(d) shows the scanning electron microscope (SEM) image of the grating antennas. The waveguide width, grating period, duty cycle, and pitch [*w*, *Λ*, *duty*, *d*] are chosen as [0.5 μm, 0.8 μm, 0.5, 1.2 μm]. The etching depth of the grating is 70 nm. The effective length of the grating antennas is about 50 μm, corresponding to the optical crosstalk of −31.2 dB. For grating antennas with larger effective length, the crosstalk needs to be significantly considered [24], [25], [26]. Figure 3(e) is the schematic diagram of the far-field measurement setup. The light source is a tunable laser with wavelength range from 1520 to 1610 nm. The polarization controller (PC) is introduced to match the polarization states of light in fiber and waveguide. The OPA chip should be placed in the center of the rotating table to avoid angle measurement errors. A feedback loop is formed of voltage control algorithm, voltage source, OPA chip, avalanche photodetector (APD) and oscilloscope. We use modified rotating element vector algorithm to compensate the initial random phase error and calibrate the far-field beam.

**Figure 4:**
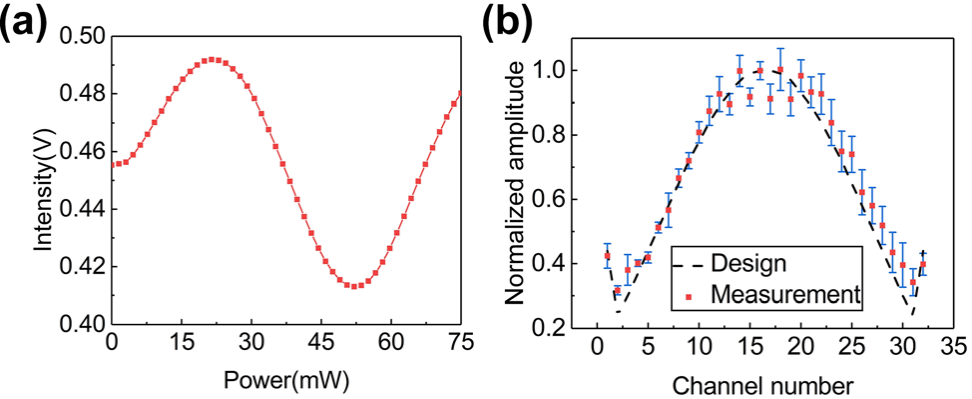
Measurement results of amplitude distribution. (a) Measured light intensity varies with different power applied on the phase shifter of the 15th channel; (b) measured amplitude distribution of the fabricated 32-channel OPA.

Firstly, we measure the amplitude distribution of the fabricated OPA based on the principle shown in part 4, Supplementary. The amplitude distribution is measured by adjusting the phase from the 1st to *N*th channel. It should be noted that the adjustment range of *δϕ*′ needs to be greater than 2π to ensure the measured results include the maximum and minimum values of the cosine function. In this work, the applied power range of the phase shifter is selected as 0–75 mW. As an example, the measured result of the 15th channel is shown in Figure 4(a). The maximum and minimum value occurs at 21.4 mW and 52.0 mW, respectively. The results of all channels are shown in Figure S10, Supplementary. Five rounds of measurements are carried out on 32 channels to minimize random errors. The measured amplitude distribution is shown in Figure 4(b), which is in good agreement with the design.

**Figure 5:**
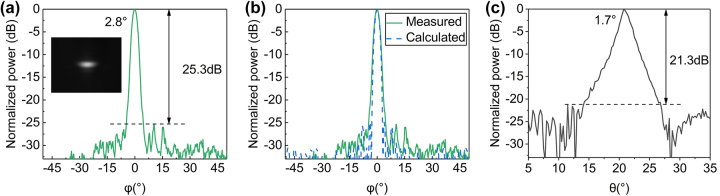
Measurement results of far-field distribution in orthogonal directions. (a) The measured far-field distribution in the azimuthal (*ϕ*) direction; (b) the measured far-field distribution as well as the far-field distribution calculated according to the measured amplitude distribution; (c) the measured far-field distribution in polar (*θ*) direction at 1550 nm wavelength. The inset in (a) is the corresponding far-field image.

After phase calibration, the far-field distribution in orthogonal directions at light wavelength of 1550 nm is shown in Figure 5. Figure 5(a) shows the cross-sectional view of the far-field beam at 0° in the azimuthal direction. The experimental SLSR is 25.3 dB, indicating an increase of 12 dB compared with conventional uniform OPA. The slight deterioration of the measured SLSR from the calculated one is mainly attributed to amplitude error, phase tuning error, and thermal crosstalk. Figure 5(b) shows the measured far-field distribution as well as the far-field distribution calculated according to the experimentally measured amplitude distribution, which is in good agreement. The SLSR in *θ* direction is 21.3 dB as shown in Figure 5(c). The measured far-field full width at half-maximum (FWHM) is 2.8° × 1.7°.

**Figure 6:**
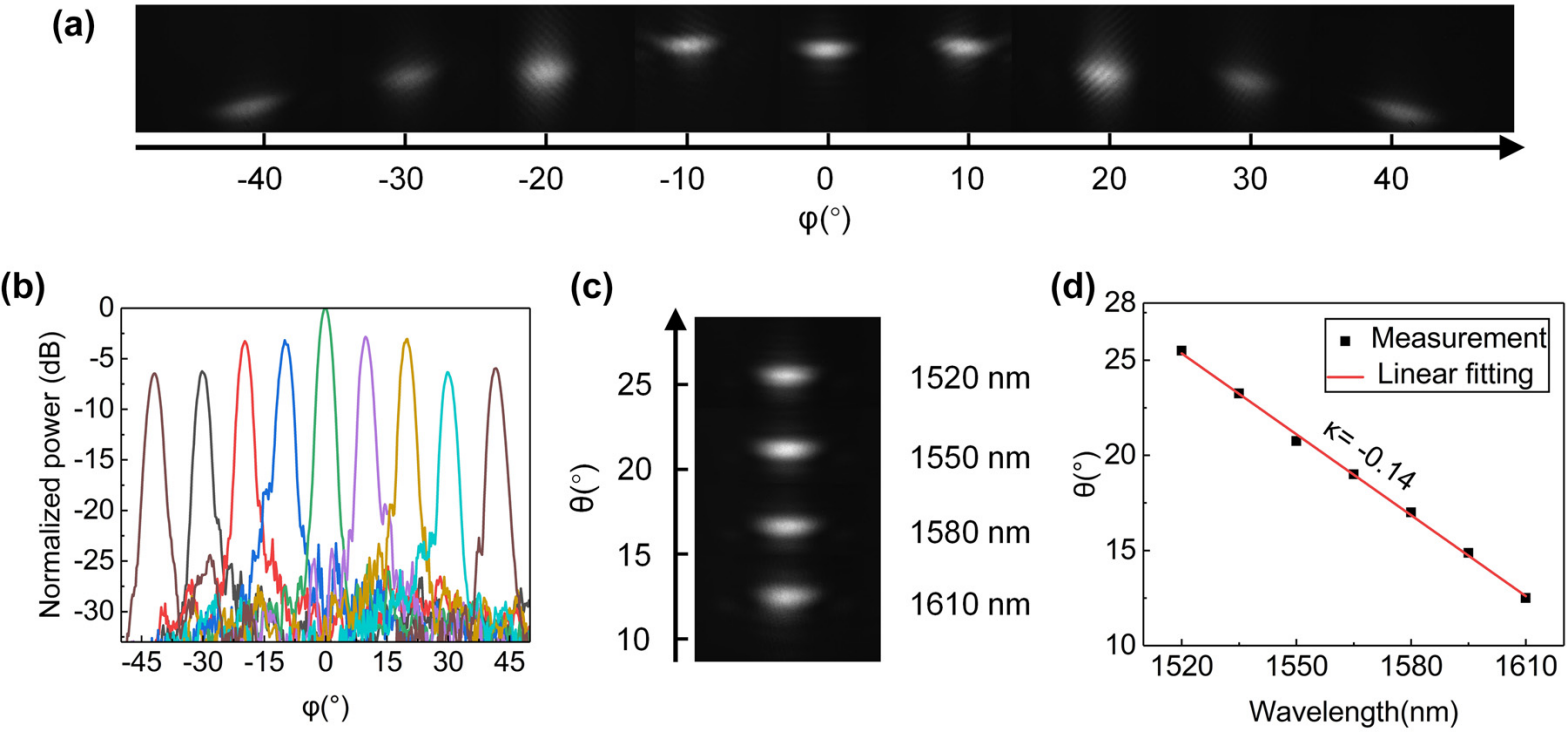
Measurement results of 2D beam steering. (a) The far-field images of beam at ±42°, ±30°, ±20°, ±10°, and 0° after calibration; (b) the measured far-field distribution in the *ϕ* direction; (c) the far-field beam images under different wavelengths; (d) the linear fitting of wavelength tuning efficiency in the *θ* direction.

Furthermore, we characterize the 2D beam steering by tuning phase shifters and wavelength. Figure 6(a) shows the images of the far-field beam at ±42°, ±30°, ±20°, ±10°, and 0°. When the beam is steered to 42°, a symmetrical beam appears at −42° degrees, corresponding to a FOV of 84° in the *ϕ* direction. The cross-sectional views of the far-field distribution are shown in Figure 6(b). The average SLSR is 19.14 dB. The beam steering in the *θ* direction is achieved by tuning the wavelength of light source from 1520 nm to 1610 nm. The far-field beam images under wavelengths of 1520 nm, 1550 nm, 1580 nm, and 1610 nm are shown in Figure 6(c), corresponding to beam steering angles of 25.5°, 20.8°,17.0°, and 12.5°, respectively. The beam steering range is from 12.5° to 25.5°. The wavelength tuning efficiency is measured to be |*κ*| = 0.14°/nm by using the linear fitting method, as shown in Figure 6(d). According to the measurement results, the FOV of the proposed OPA is 84° × 13°.

**Figure 7:**
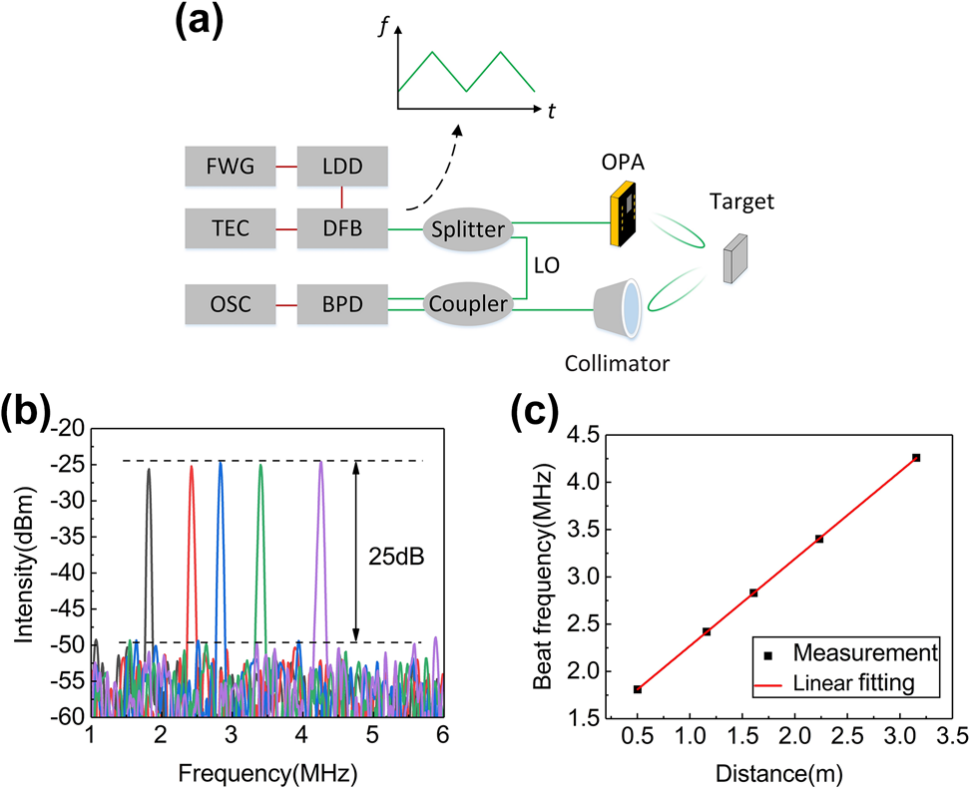
Free-space LIDAR range measurement based on the proposed OPA. (a) Schematic of FMCW ranging system; (b) measured FMCW beat signal FFT spectra with target placed in five different distances; (c) the linear fitting of measured FMCW beat frequency depending on distance.

Using the proposed OPA as the transmitter (TX) and a collimator as the receiver (RX), the schematic of our FMCW ranging system is shown in Figure 7(a). A laser diode driver (LDD), modulated by optimized waveform signal generated by a function waveform generator (FWG), drives the distributed feedback laser (DFB) to generate a linear frequency chirp signal, as shown in Figure 7(a). The repetition rate is 2 kHz and the chirp bandwidth is 34.6 GHz. A temperature controller (TEC) is introduced to keep the DFB operating at 25 °C. The signal is divided into two parts with splitting ratio of 99: 1. The minor portion is used as the local oscillator (LO). The major portion emits to free space at 0° in the azimuthal direction through the proposed OPA. The light, reflected by the target, couples to the fiber by the collimator and optically mixes with LO using the coupler. A balanced photodetector (BPD) converts the optical signal into electric signal, which is recorded by the oscilloscope (OSC). The FMCW beat frequency is expressed as:

(4) $$\begin{array}{c} f_{B}=2Bf_{R}\cdot \frac{L_{TX}+L_{RX}+L_{0}}{c}\\ =\frac{4Bf_{R}}{c}\cdot L+f_{B0} \end{array}$$

where *B* is the chirp bandwidth, *c* is velocity of light, *f*_*R*_ is the repetition rate, and *L*_*TX*_ (*L*_*RX*_) is the distance from TX (RX) to the target. TX and RX are placed closely next to each other. As the target is far enough away from them, *L*_*TX*_ and *L*_*RX*_ are approximately equal, both denoted by *L*. *L*_0_ is the optical path difference between LO light and the signal light due to the fiber, OPA chip, and collimator, corresponding beat frequency of *f*_*B*0_. The target is placed in five distances (0.50, 1.16, 1.61, 2.23, and 3.16 m). The measured fast Fourier transform (FFT) spectra of beat signals are shown in Figure 7(b), under the beat frequency of 1.81, 2.42, 2.83, 3.40, and 4.26 MHz. The signal-to-noise ratios (SNRs) are all >25 dB. The power of DFB is measured to be 8.4 dBm, and the coupling loss of the grating coupler is 4.3 dB. The emission efficiency of grating antennas is about 3 dB. If further increasing the power of DFB, improving the coupling efficiency [27], [28], [29], and introducing the unidirectional grating antennas [30], [31], [32], the SNR can be further increased, which is conducive to increasing the maximum detection range [33]. Figure 7(c) is the linear fitting of measured FMCW beat frequency depending on distance with slope of 0.92 MHz/m and *f*_*B*0_ of 1.35 MHz.

## 4 Conclusions

In summary, we propose and demonstrate an OPA with low sidelobe level. The beam splitter tree is constructed with ARPSs, of which the distribution form is flexible and has large fabrication tolerance. The Dolph-Chebyshev amplitude distribution is chosen due to its good balance between beam width and sidelobe level. The measured amplitude distribution of the fabricated OPA is in good agreement with the design. The experimental results indicate that the SLSRs in *ϕ* = 0° is 25.3 dB and the average SLSR within whole FOV is 19.1 dB. The measured FOV is 84 × 13° with FWHM of 2.8 × 1.7°. In addition, we combine the fabricated OPA chip with FMCW based ranging system and realize precise ranging experimentally. To the best of our knowledge, this is the first demonstration of OPA utilizing Dolph-Chebyshev amplitude distribution based on ARPSs. In Table 1, the key performances of several reported OPAs are summarized. The proposed OPA in our work shows an ultra-high SLSR. The beam divergence can be further decreased by increasing the number of channels and reducing the grating strength [6], [8].

**Table 1:** Summary of integrated OPAs.

Ref.	Type of amplitude distribution	Power splitter structure	FOV (°)	FWHM (°)	SLSR^a^ (dB)
[10]	Uniform	3 dB MMI based splitter tree	70 × 6	0.15 × 0.08	7.5
[13]	Gaussian	Star coupler	22 × 28	0.78 × 0.02	∼16
[11]	Gaussian	Star coupler	140 × 13.5	2.1 × 0.08	19 (±40°) 13.2 (±70°)
[14]	Gaussian	Cascaded DCs	25 × 13.2	0.31 × 0.07	15.1 (0°)
This work	Dolph–Chebyshev	ARPS based splitter tree	84 × 13	2.8 × 1.7	25.3 (0°) 19.1 (avg)

^a^SLSRs in azimuthal (*ϕ*) direction.

## Supplementary Material

Supplementary Material Details
