# Supplementary material for: Low sidelobe silicon optical phased array with Chebyshev amplitude distribution
Source: Nanophotonics. 2024 Jan 22;13(3):263–9. doi: 10.1515/nanoph-2023-0507 (PMC11501257; doi:10.1515/nanoph-2023-0507)
Supplement: Supplementary file 1 — Supplementary Material Details [file j_nanoph-2023-0507_suppl_001.docx]

Supplementary for:

Low Sidelobe Silicon Optical Phased Array with Chebyshev Amplitude Distribution

*Shi Zhao, Daixin Lian, Wenlei Li, Jingye Chen,^1^ Daoxin Dai, and Yaocheng Shi**

State Key Laboratory for Modern Optical Instrumentation, Center for Optical and Electromagnetic Research, International Research Center for Advanced Photonics, Ningbo Innovation Center, College of Optical Science and Engineering, Zhejiang University, China.

* Address correspondence to: yaocheng@zju.edu.cn

^1^ jingyechen@zju.edu.cn

**Contents**

1. Calculation of Dolph-Chebyshev amplitude distribution
2. Design and tolerance analysis of ARPSs
3. Parameters of beam splitter tree
4. Method of amplitude distribution measurement
5. Analysis of thermal crosstalk

**1. Calculation of Dolph-Chebyshev amplitude distribution**

The Chebyshev polynomial is expressed as [S1]:

 (S1)

The recurrence formula is:

 (S2)

The Chebyshev polynomial has the following properties: 1) *T_n_*(*x*) is a *n*-th order polynomial of *x*; 2) even (odd) order polynomials contain only even (odd) terms; 3) *T_n_*(*x*) oscillates between ±1 when |*x*|<1; 4) |*T_n_*(*x*)| rises monotonically with hyperbolic type when |*x*|≥1. The amplitude distribution is calculated based on Chebyshev polynomial and its properties.

For an OPA with 2*M* channels, we define amplitude distribution center symmetry and the reference point as the center of the array. The array factor (*Ar*) with beam steered to *φ*_0_ could be express as follows:

$Ar\left(u\right)=\sum_{m=1}^{M} 2E_{m}\cos\left[\left(2m-1\right)\frac{u}{2}\right]$ (S3)

where *u*=*k*_0_*d*(sin*φ*-sin*φ*_0_), *d* is the grating antenna pitch, and *k*_0_ is 2π/*λ*. Let *x*=*b*cos(*u*/2), where *b*>1. According to equation S1, the equation S3 can be written as:

$\begin{matrix} Ar\left(x\right)=\sum_{m=1}^{M} 2E_{m}T_{2m-1}\left(\frac{x}{b}\right) \\ =2E_{1}\left(\frac{x}{b}\right)+2E_{2}\left[4\left(\frac{x}{b}\right)^{3}-3\left(\frac{x}{b}\right)\right]+...+2E_{M}T_{2M-1}\left(\frac{x}{b}\right) \end{matrix}$ (S4)

The SLSR and channel number in our work is set 30 dB and 32 (*M*=16). *b* is calculated to be 1.009, accorder following equation:

 (S5)

*Ar*(*x*) is a polynomial containing *x*, *x*^3^, …, *x*^2^*^M^*^-1^, which is similar to *T*_2_*_M_*_-1_(*x*). Let *Ar*(*x*)= *T*_2_*_M_*_-1_(*x*). Then matching the similar terms of *Ar*(*x*) and *T*_2_*_M_*_-1_(*x*), the amplitude distribution with given SLSR could be obtained by solving the linear equations. The calculated amplitude distribution is shown in Table S1. The calculated far-field amplitude distribution with beam steered to 30° is shown in Fig. S1. *x* equals to *b* when *φ=*30° and -53°, corresponding to the 0th and -1st order grating lobes. At other *φ* than the grating lobes, the amplitude oscillates between 0 and 1, as shown in the inset.

The SLSR in our work is set 30 dB. The higher SLSR, e.g., 40 dB shown in Fig. S2, could also be achieved.

**Table S1**: Calculated amplitude distribution in this work.

| Channel | 1 | 2 | 3 | 4 | 5 | 6 | 7 | 8 |
| --- | --- | --- | --- | --- | --- | --- | --- | --- |
| Amplitude | 0.44 | 0.24 | 0.30 | 0.37 | 0.44 | 0.51 | 0.58 | 0.65 |
| Channel | 9 | 10 | 11 | 12 | 13 | 14 | 15 | 16 |
| Amplitude | 0.72 | 0.78 | 0.84 | 0.89 | 0.93 | 0.97 | 0.99 | 1.00 |
| Channel | 17 | 18 | 19 | 20 | 21 | 22 | 23 | 24 |
| Amplitude | 1.00 | 0.99 | 0.97 | 0.93 | 0.89 | 0.84 | 0.78 | 0.72 |
| Channel | 25 | 26 | 27 | 28 | 29 | 30 | 31 | 32 |
| Amplitude | 0.65 | 0.58 | 0.51 | 0.44 | 0.37 | 0.30 | 0.24 | 0.44 |


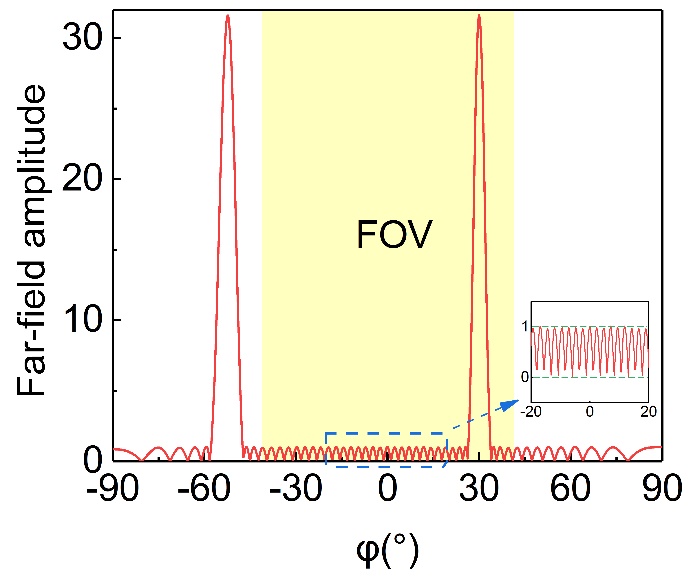


**Figure S1:** The calculated far-field amplitude distribution of Dolph-Chebyshev array.


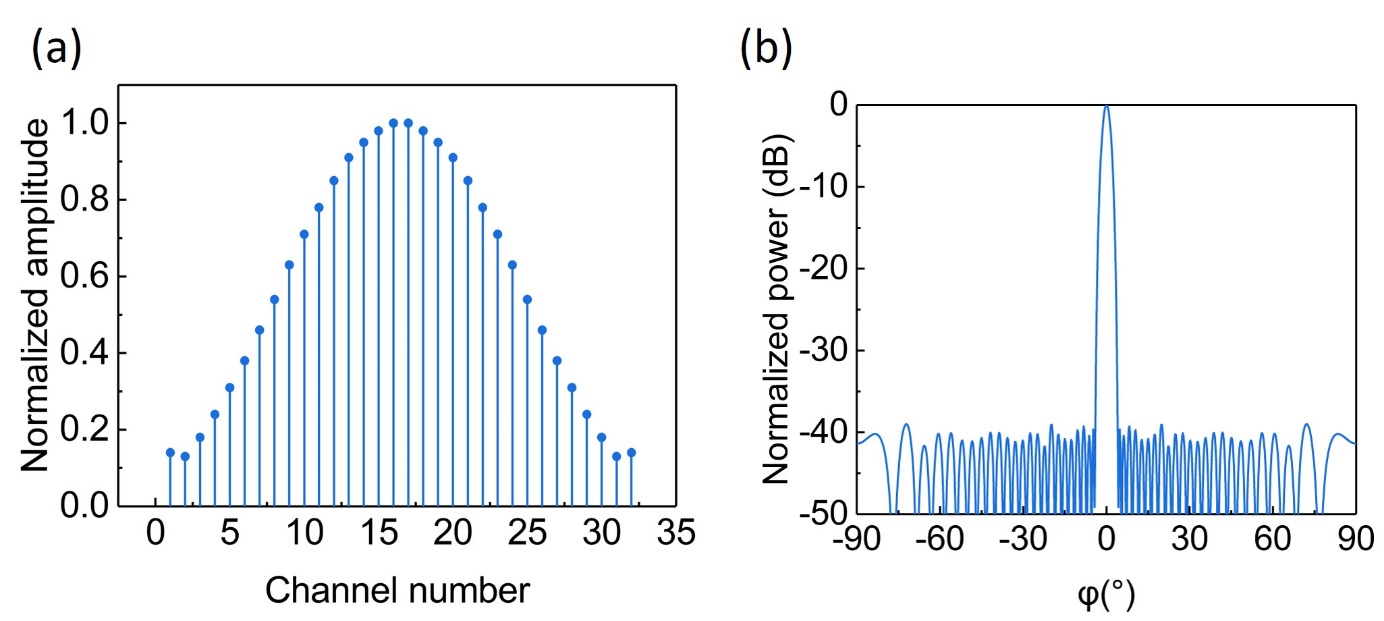


**Figure S2:** (a) Dolph-Chebyshev amplitude distribution; (b) the far-field pattern of (a) with SLSR of 40 dB.

**2. Design and tolerance analysis of ARPSs**

The schematic of the ARPS is shown in Fig. S3. The contours of the MMI section are two types of Bezier curves: Bezier curve A and Bezier curve B. The arbitrary power splitting ratios are obtained by keeping Bezier curve A unchanged and adjusting Curve point (B2) of Bezier curve B to introduce structure asymmetry. Here, we denote the asymmetry (*Asym*) of MMI as:

$Asym\text{ = }L_{\text{B2}-\text{B3}}/L_{\text{mmi}}$ (S6)

the power splitting ratio (*PS*) as:

$PS\text{ = }\frac{T_{\text{Output}1}}{T_{\text{Output}1}+T_{\text{Output2}}}$ (S7)

and the excess loss (*EL*) as:

$EL=-10\log_{10}\left(T_{\text{Output}1}+T_{\text{Output2}}\right)$ (S8)

The three-dimensional finite-difference time-domain (3D-FDTD) method is performed to simulate the devices. The calculated *PS*s with different *L*_mmi_ and *Asym* are shown in Fig. S4(a). The calculated wavelength sensitivity (*WS*s=max(*PS*)-min(*PS*)) and maximum *EL*s with the wavelength from 1500 nm to 1600 nm are shown in Figs. S4(b) and S4(c). To balance the performance of wavelength sensitivity and excess loss, the [*Asym*, *L*_mmi_] parameters are selected on the blue dot line (shown in Fig. S4).


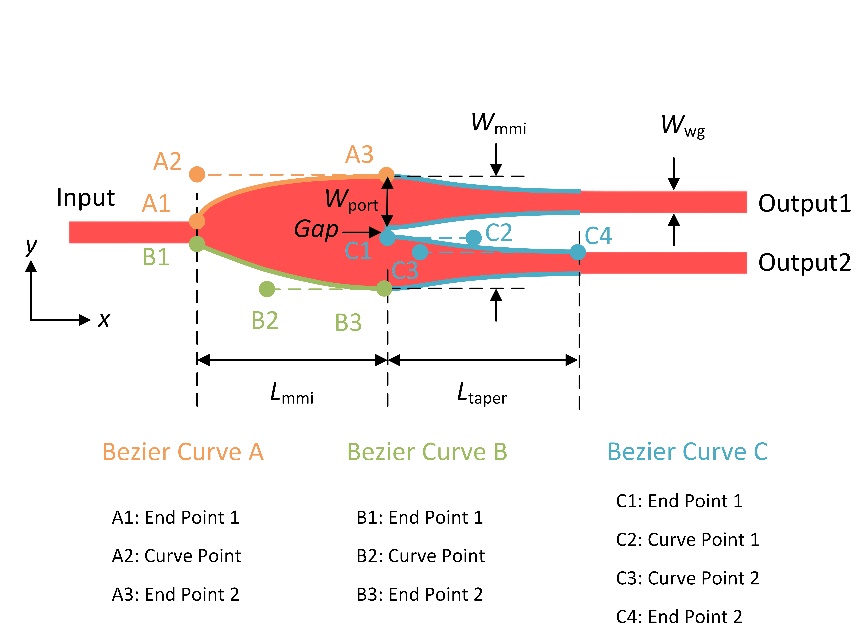


**Figure S3:** Schematic of the arbitrary ratio power splitter.


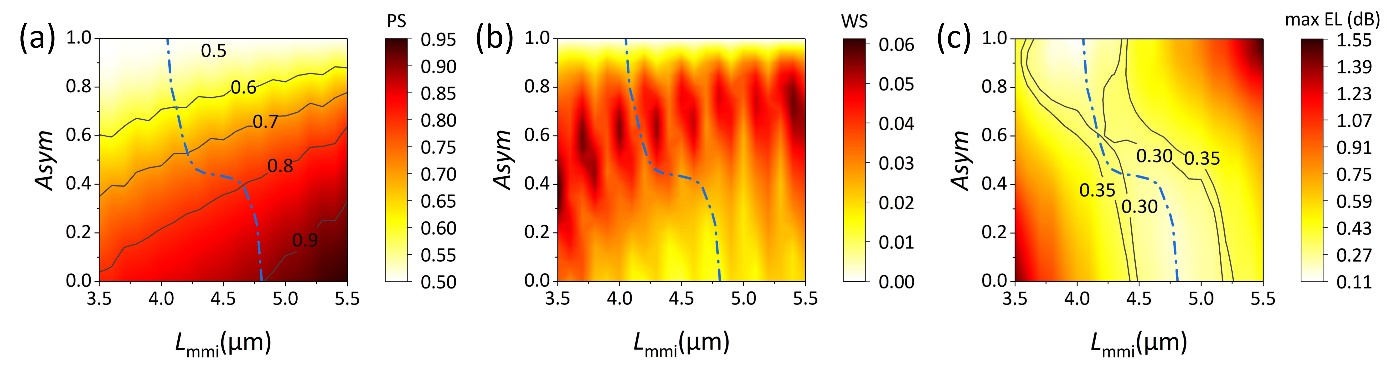


**Figure S4:** Calculated (a) power splitting ratio, (b) wavelength sensitivity, and (c) excess loss of the ARPSs with different parameters.

The calculated *PS*s and *EL*s spectra are shown in Fig. S5. The *PS*s at 1550 nm are 0.5, 0.6, 0.7, 0.8, and 0.9, corresponding to [*Asym*, *L*_mmi_] of [1, 4.05 μm], [0.71, 4.12 μm], [0.53, 4.21 µm], [0.40, 4.66 µm], and [0.01, 4.81 µm]. The maximum *WS* is 0.033, appearing in *PS*=0.6. In Fig. S5, we also calculated the *PS*s and *EL*s with device widths variation *∆W* = ±40 nm. The *∆PS*s are less than 0.065, and the *EL*s are all below 0.35 dB. The overall scaling of the device is an extremely special case. To be more realistic, the normal distribution is used to simulate the random drift of width after width calibration. We assume that there are no correlations between the parameters and the distributions of each parameter are *W*_wg_ ~ *N* (500nm, 13.3nm^2^), *Gap* ~ *N* (200nm, 13.3nm^2^), and *W*_mmi_ ~ *N* (2500nm, 13.3nm^2^). The +3 Sigma and -3 Sigma are +40 nm and -40 nm respectively. The Monte Carlo analysis can be useful for assessing statistical variations of multiple parameters on device performance, as well as circuit elements on overall circuit performance. According to Fig. S5, *∆PS* of ARPS is maximum when *PS* = 0.7, which is selected for further analysis. The simulation time for one ARPS is about 2’20”. In Monte Carlo analysis, the number of trails is set to be 200, taking about 8 hours. The structure parameters of different trails index are shown in Figs. S6(a) to S6(c) and the counts of different structure parameters are shown in Figs. S6(d) to S6(f). Figures S6(g) to S6(l) show the correlations of *PS* (or *EL*) with different structure parameters. The *∆PS*s are less than 0.055, and the *EL*s are all below 0.37 dB. From Figs. S6(i) and S6(k), it could be found that the *∆PS* is mainly dependent on the variation of waveguide width *W*_wg_, and the *∆EL* is mainly dependent on the variation of gap width *Gap*. The width of the MMI region near the waveguide changes rapidly, while the width of the MMI region near the output ports changes slowly, so the influence of waveguide width changes on the symmetry of the MMI region is dominant. An increase in *Gap* will increase reflection and introduce greater loss. Those could well explain the trend in Fig. S6.


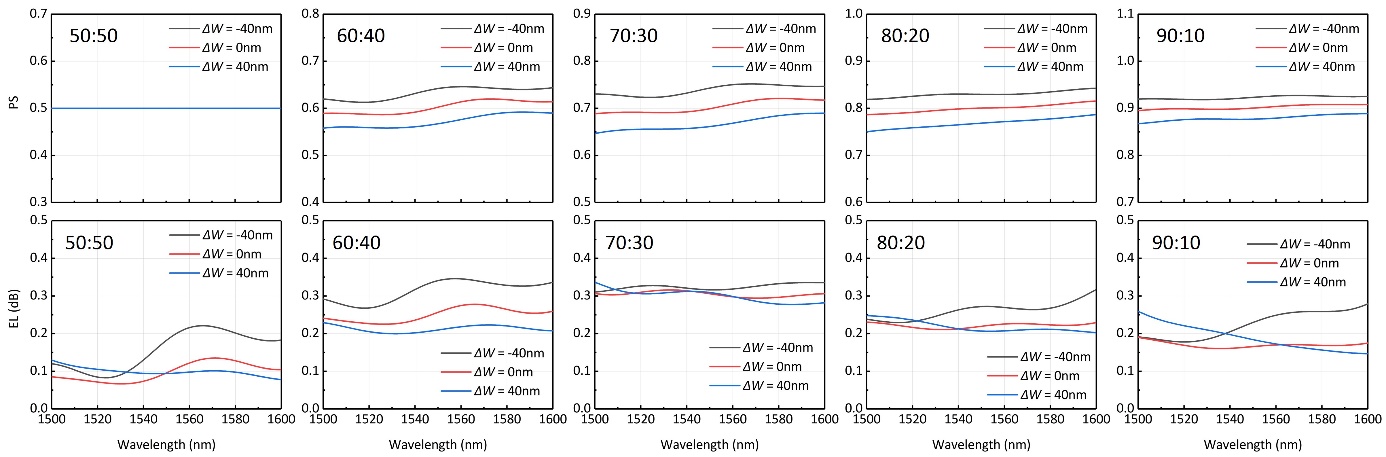


**Figure S5:** Calculated fabrication tolerance of ARPSs.


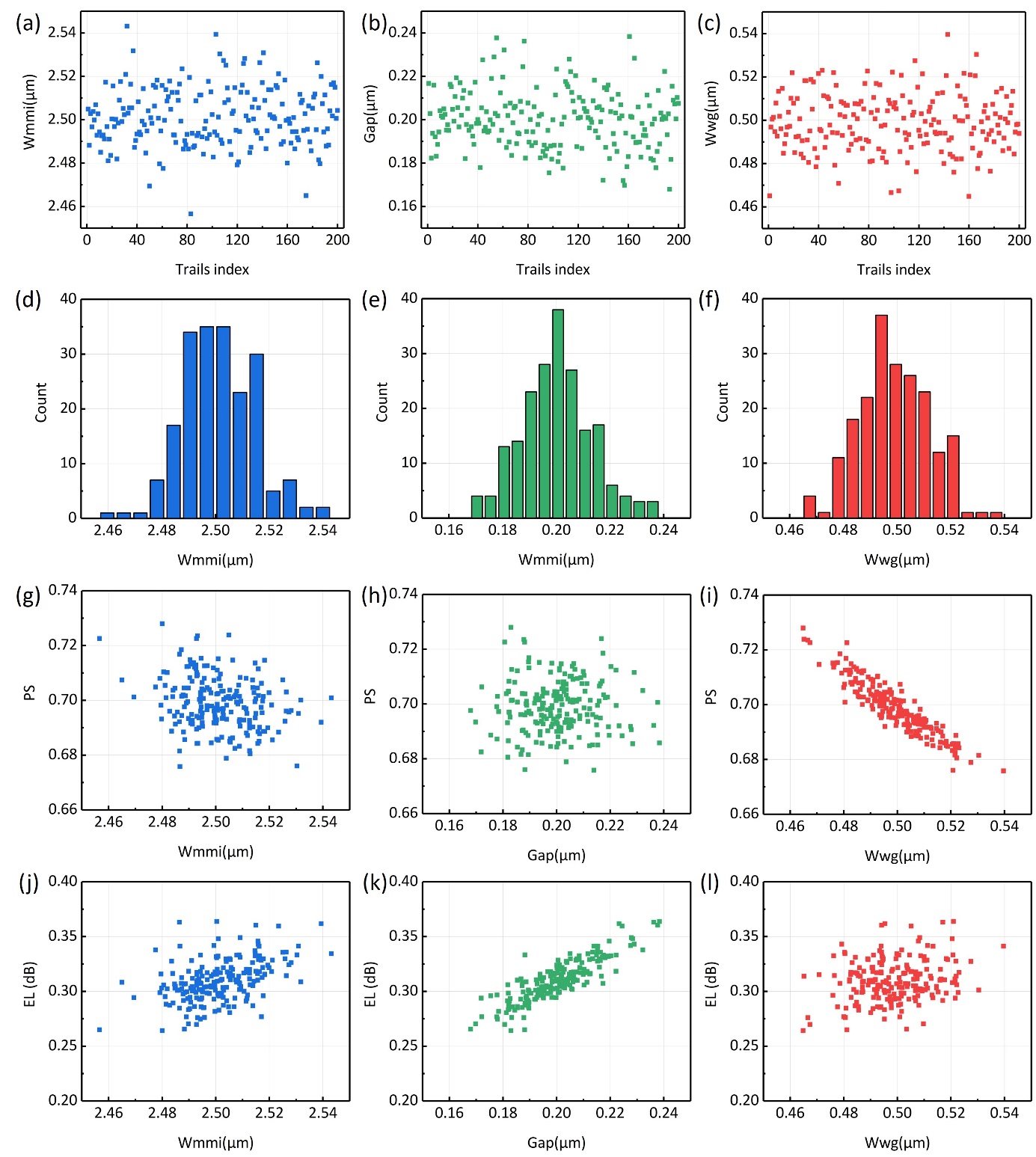


**Figure S6:** Monte Carlo analysis for fabrication tolerance of ARPSs. (a)-(c): structure parameters of different trails index; (d)-(f): counts of different structure parameters; (g)-(i): correlation of *PS* with different structure parameters; (j)-(l): correlation of *EL* with different structure parameters.

The thermo-optic coefficients of Si and SiO_2_ are set to be 1.8×10^-4^ and 8.6×10^-6^ [S2-S3]. The thermal sensitivity of ARPS is also characterized as shown in Fig. S7. The *PS* and *EL* are almost invariable with temperature. As shown in the enlarged views of *PS*=0.7, the *∆PS* is less than 0.01 and the *∆EL* is less than 0.01 dB with *∆T* = ±50 K (from -23 to 77 ℃), which can be well stabilized by TEC in the experiment.


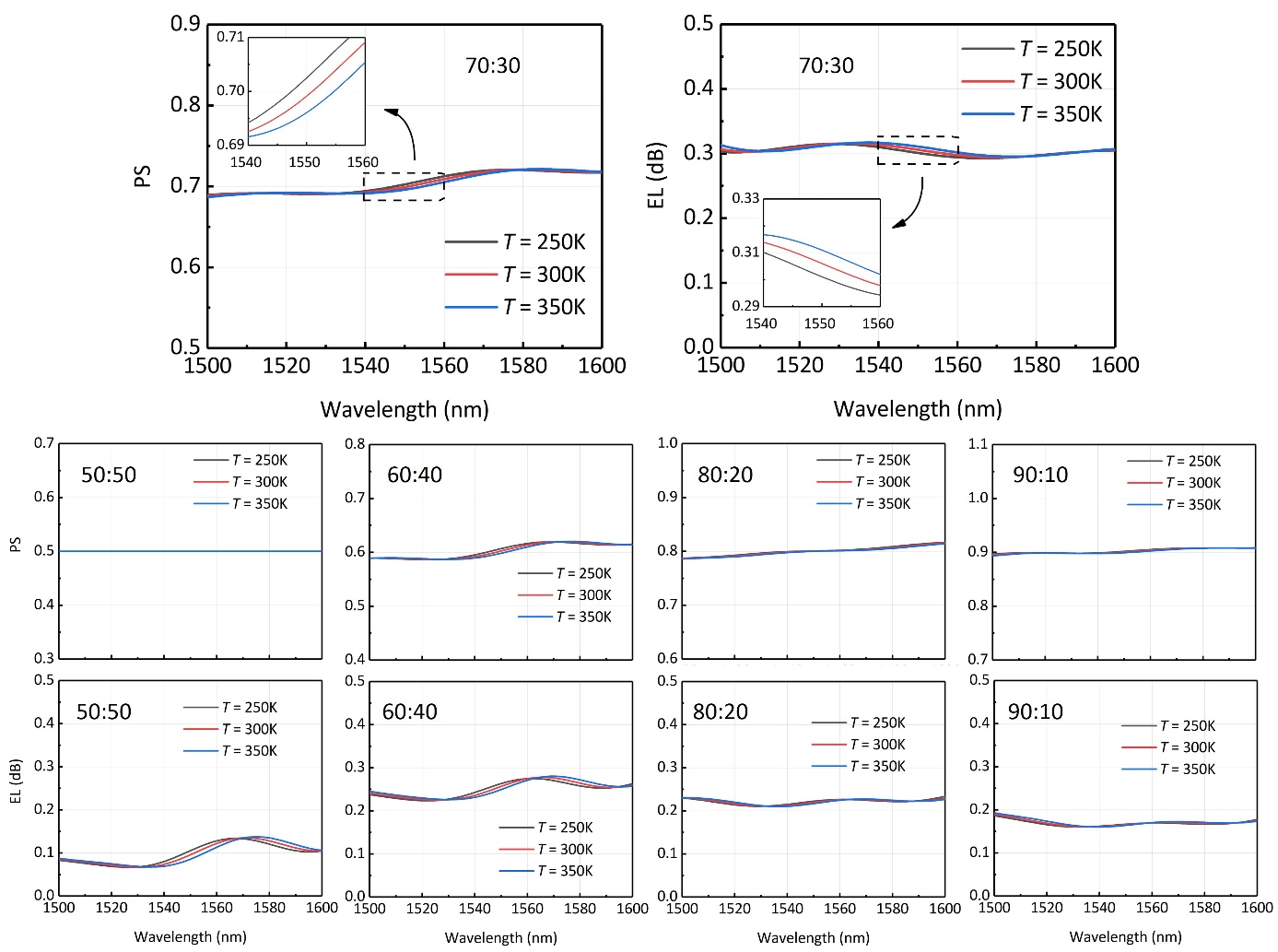


**Figure S7:** Calculated thermal sensitivity of ARPSs.

**3. Parameters of beam splitter tree**

**
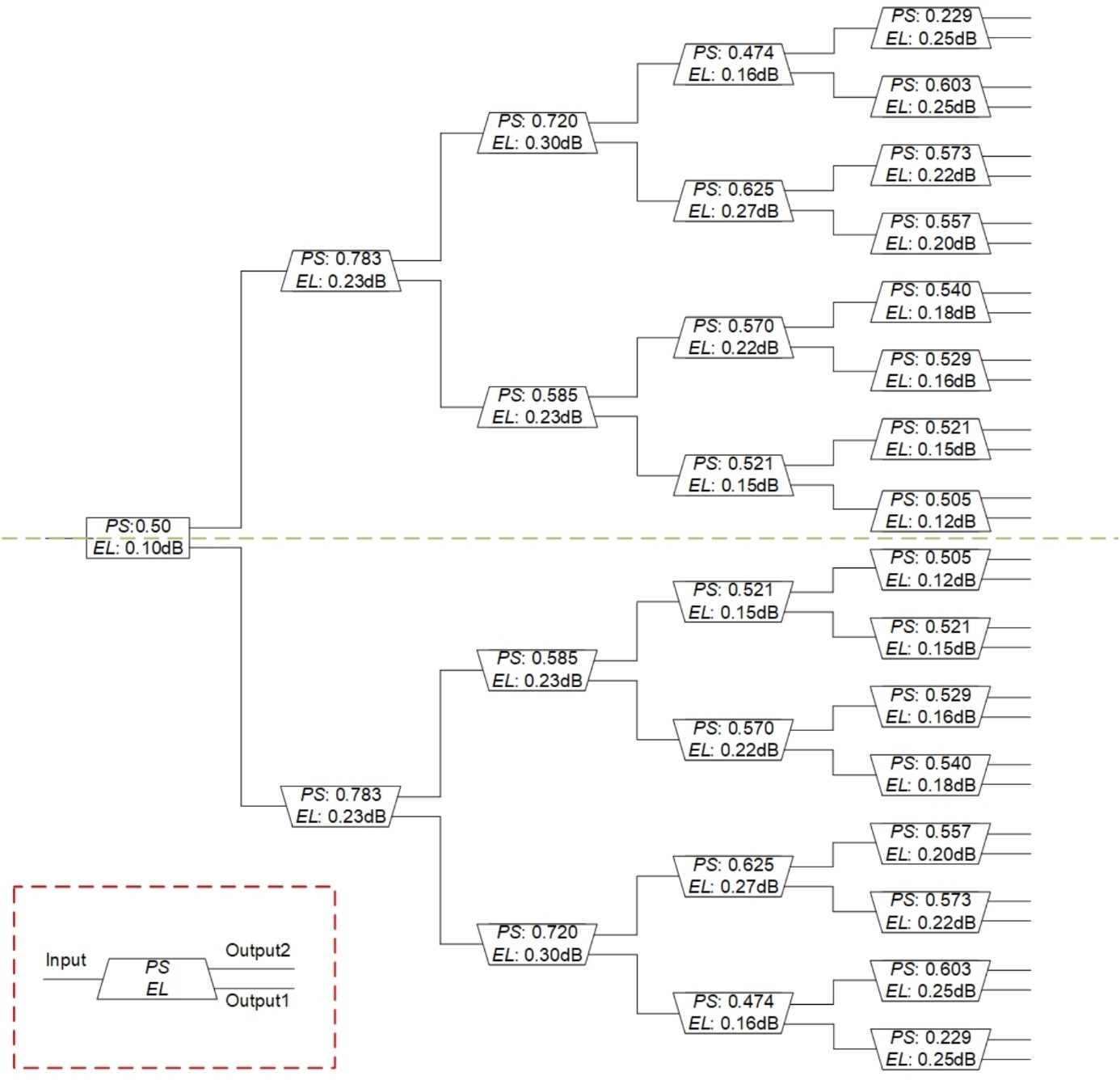
**

**Figure S8:** The *PS* and *EL* of each ARPS in the beam splitter tree. The inset illustrates the definition of ports.

According to the inverse calculation method proposed in this work, the calculated power splitting ratio (*PS*s) and corresponding excess losses (*EL*s) of ARPSs in the splitter tree are shown in Fig. S8. The definition of ports is shown in the red dotted box. The ARPSs structure parameters could be find in **Part 2**. The amplitude distribution is symmetrical as shown in Tab. S1. Hence, the splitter tree is symmetrical about the green dotted line, shown in Fig. S8.

**4. Method of amplitude distribution measurement**

The near-field distribution of OPA can be expressed as:

 (S9)

where *f*(*x*-*nd*) is the normalized near-field distribution of the *n*-th antenna. *E_n_* and *ϕ_n_* are the amplitude and phase of the *n*-th channel. *d* is the grating antenna pitch. *ϕ_n_* includes the random errors caused by manufacturing and the phase shift introduced by phase shifters. The far-field distribution is the Fourier transform of the near-field [S4], expressed by the following formula:

 (S10)

where *F*(*k_x_*) is the Fourier transform of *f*(*x*). The angular spatial frequency *k_x_* is expressed as (2π/*λ*)sin(*φ*), where *λ* is the working wavelength. Hence, the far-field distribution is a function of angle *φ*. When avalanche photodetector (APD) is placed in *φ*_0_, OPA is coherence enhanced in this direction after calibration, that is, *ϕ_n_*-*ndk_x_*=2*m*π+*ϕ’*, *m*=0, ±1, ±2, …. Then, adjusting the phase of the *i*-th channel and keeping the phase of the remaining *N*-1 channels unchanged, the complex amplitude and intensity at *φ*_0_ direction are shown as follows:

 (S11)

 (S12)

where *δϕ’* is the phase difference between the adjusted channel and unchanged channels, as shown in Fig. S9. We use the *E_fix_* to express the sum of the amplitude of the *N*-1 unchanged channels. From the formula S11, it can be found that the light intensity at *φ*_0_ direction changes in the form of cosine with *δϕ’*. The light intensity reaches the maximum |*F*(*φ*_0_)|^2^·$(\sum_{n=1}^{N} E_{n})$^2^ (blue point shown in Fig. S9) when *δϕ’*=2*m*π. The intensity reaches the minimum |*F*(*φ*_0_)|^2^·$(\sum_{n=1}^{N} E_{n}-2E_{i})$^2^ (origin point shown in Fig. S9) when *δϕ’*=(2*m*+1)π, where *m*=0, ±1, ±2, …. Therefore. *E_i_* is proportional to {Max[*E*(*φ*_0_)]-Min[*E*(*φ*_0_)]}/2.


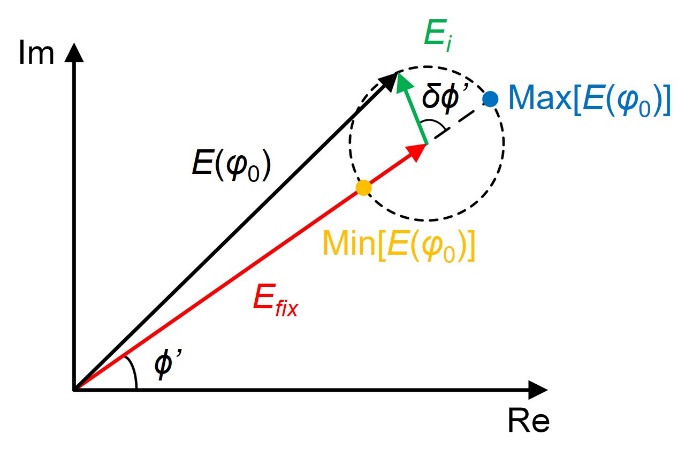


**Figure S9:** Complex amplitude at *φ*_0_ direction represented on the complex plane.

The measured light intensity variations of all channels (round 2) are shown in Fig. S10. The light intensity at *φ*_0_ direction is collected by an APD and recorded by an oscilloscope. Hence, we express the light intensity in unit of mV. The maximum values of all channels are almost the same, which are proportional to $(\sum_{n=1}^{N} E_{n})$^2^. The minimum values, related to the light intensity of each channel, are proportional to $(\sum_{n=1}^{N} E_{n}-2E_{i})$^2^. The measured light intensity varies with different power is basically in the form of cosine, which agrees well with the above analysis.


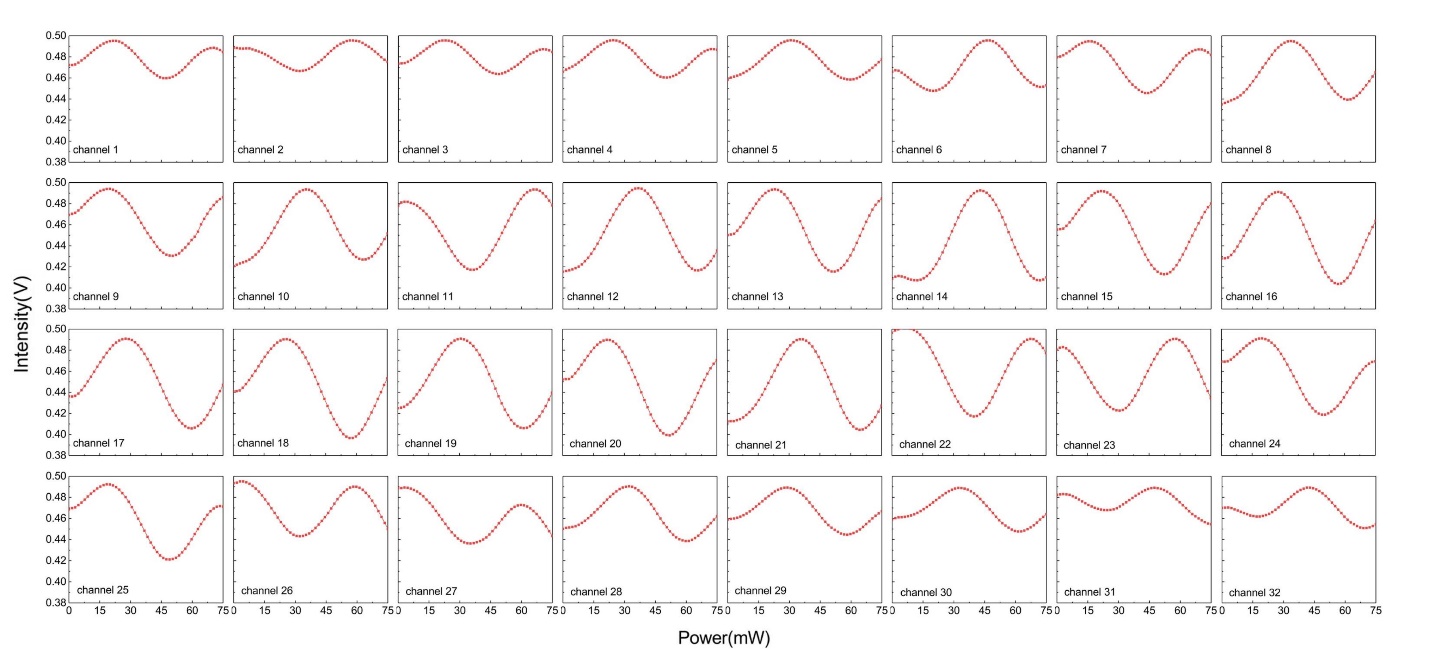


**Figure S10:** Measured light intensity varies with different power applied on the phase shifter of all channels.

**5. Analysis of thermal crosstalk**

The distance of adjacent phase shifters is set as 40 μm. The thermal conductivities of silicon and silica are set to be 148 and 1.38 W/(m·K), and the heat convection coefficient between air and silica is set to be 5 W/(m^2^ ·K). To characterize thermal crosstalk, we apply 50mW of power to WG-B and 0 mW to WG-A and WG-C, as shown in Fig. S11(a). Fig. S11(c) shows the thermal distribution of the waveguide layer. The thermal crosstalk between the adjacent waveguides is below -20.2 dB. When the phase shift of WG-B is 2π rad, the phase errors, introduced by thermal crosstalk, of WG-A and WG-C is 0.019π rad.


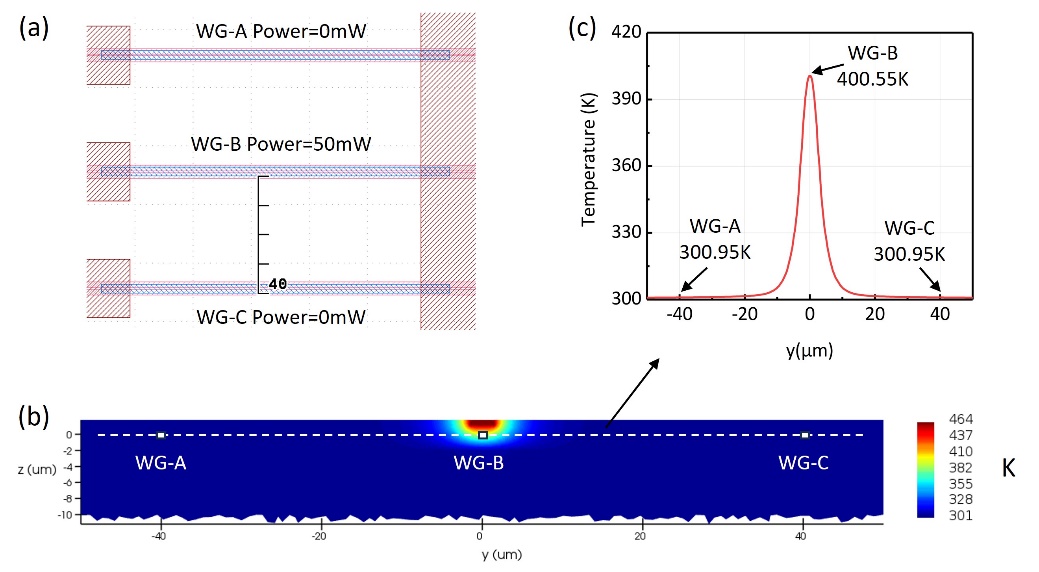


**Figure S11:** (a) Layout of phase shifter array; (b) cross-section thermal distribution, the dashed white line (*Z* =0 µm) is the layer where the waveguide is located; (c) thermal distribution with *Z*=0 µm.

**References:**

[S1] G. T. Freitas de Abreu and R. Kohno, "A modified Dolph-Chebyshev approach for the synthesis of low sidelobe beampatterns with adjustable beamwidth," *IEEE Trans. Antennas Propag.*, vol. 51, no. 10, pp. 3014-3017, 2003.

[S2] S. Liu, J. Feng, Y. Tian, H. Zhao, L. Jin, B. Ouyang, J. Zhu, and J. Guo, "Thermo-optic phase shifters based on silicon-on-insulator platform: state-of-the-art and a review," *Front. Optoelectron.*, vol. 15, no. 1, 2022.

[S3] A. Arbabi and L. L. Goddard, "Measurements of the refractive indices and thermo-optic coefficients of Si_3_N_4_ and SiO(_x_) using microring resonances," *Opt. Lett.*, vol. 38, no. 19, pp. 3878-81, 2013.

[S4] M. J. R. Heck, "Highly integrated optical phased arrays: photonic integrated circuits for optical beam shaping and beam steering," *Nanophotonics*, vol. 6, no. 1, pp. 93-107, 2017.
